# Diaqua­hexa-μ_2_-dichloro­acetato-μ_3_-oxido-tetra­hydro­furan­diiron(III)manganese(II)

**DOI:** 10.1107/S1600536809054518

**Published:** 2009-12-24

**Authors:** Omid Sadeghi, Mostafa M. Amini, Seik Weng Ng

**Affiliations:** a Department of Chemistry, General Campus, Shahid Beheshti University, Tehran 1983963113, Iran; b Department of Chemistry, University of Malaya, 50603 Kuala Lumpur, Malaysia

## Abstract

In the oxido-centered title compound, [Fe_2_Mn(C_2_HCl_2_O_2_)_6_O(C_4_H_8_O)(H_2_O)_2_], the central O atom is linked to three metal atoms, which are themselves each linked to four dichloro­acetate anions, and is in a triangular configuration. Two of the metal atoms are each coordinated by a water mol­ecule, whereas the third is coordinated by a tetra­hydro­furan mol­ecule. In the crystal, adjacent mol­ecules are linked by O—H⋯O and O—H⋯Cl hydrogen bonds across centers of inversion, generating a hydrogen-bonded chain along the *c* axis. The Mn^II^ atoms are disordered with respect to the Fe^III^ atoms, and the same metal site is occupied by 1/3Mn + 2/3Fe.

## Related literature

For aqua­bis(tetra­hydro­furan)hexa­kis(trifluoro­acetato)(*μ*_3_-oxido)*M*(II)diiron(III) (*M* = copper, zinc), see: Amini *et al.* (2004*a* ▶,*b* ▶).
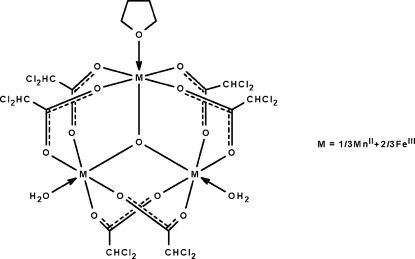


## Experimental

### 

#### Crystal data

[Fe_2_Mn(C_2_HCl_2_O_2_)_6_O(C_4_H_8_O)(H_2_O)_2_]
*M* *_r_* = 1058.34
Triclinic, 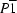

*a* = 9.380 (1) Å
*b* = 13.316 (1) Å
*c* = 15.432 (1) Å
α = 90.131 (1)°
β = 100.067 (1)°
γ = 97.677 (1)°
*V* = 1880.1 (2) Å^3^
*Z* = 2
Mo *K*α radiation
μ = 2.01 mm^−1^
*T* = 295 K
0.35 × 0.15 × 0.15 mm

#### Data collection

Bruker SMART APEX diffractometer
Absorption correction: multi-scan (*SADABS*; Sheldrick, 1996 ▶) *T*_min_ = 0.540, *T*_max_ = 0.753
15425 measured reflections
8543 independent reflections
5788 reflections with *I* > 2σ(*I*)
*R*_int_ = 0.025

#### Refinement

*R*[*F* ^2^ > 2σ(*F* ^2^)] = 0.063
*wR*(*F* ^2^) = 0.207
*S* = 1.03
8543 reflections
440 parameters
35 restraints
H atoms treated by a mixture of independent and constrained refinement
Δρ_max_ = 1.64 e Å^−3^
Δρ_min_ = −0.90 e Å^−3^

### 

Data collection: *APEX2* (Bruker, 2008 ▶); cell refinement: *SAINT* (Bruker, 2008 ▶); data reduction: *SAINT*; program(s) used to solve structure: *SHELXS97* (Sheldrick, 2008 ▶); program(s) used to refine structure: *SHELXL97* (Sheldrick, 2008 ▶); molecular graphics: *X-SEED* (Barbour, 2001 ▶); software used to prepare material for publication: *publCIF* (Westrip, 2010 ▶).

## Supplementary Material

Crystal structure: contains datablocks global, I. DOI: 10.1107/S1600536809054518/ci2991sup1.cif

Structure factors: contains datablocks I. DOI: 10.1107/S1600536809054518/ci2991Isup2.hkl

Additional supplementary materials:  crystallographic information; 3D view; checkCIF report

## Figures and Tables

**Table 1:** Hydrogen-bond geometry (Å, °)

*D*—H⋯*A*	*D*—H	H⋯*A*	*D*⋯*A*	*D*—H⋯*A*
O1*w*—H11⋯O3^i^	0.85 (1)	2.01 (3)	2.809 (6)	158 (5)
O2*w*—H22⋯O8^ii^	0.85 (1)	2.06 (4)	2.821 (5)	149 (7)
O2W—H21⋯O10^ii^	0.84 (6)	2.19 (7)	2.950 (6)	150 (6)
O1W—H12⋯Cl1^i^	0.85 (3)	2.47 (4)	3.288 (4)	160 (6)

Symmetry codes: (i) 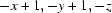; (ii) 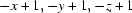.

**Fig. 1.**
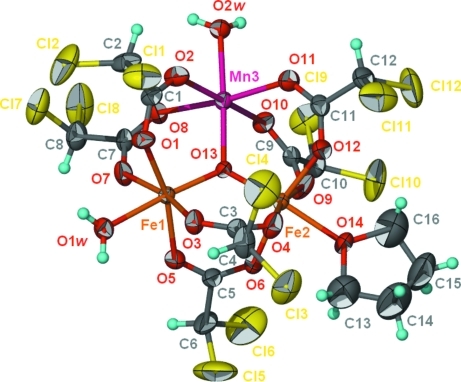
Displacement ellipsoid plot (Barbour, 2001) of Fe2MnO(H2O)2(C4H8O)(C2HO2Cl2)6; ellipsoids are drawn at the 50% probability level and H atoms of arbitrary radius.

## supplementary crystallographic information

### Experimental

Sodium bicarbonate (4.12 g, 49 mmol) was dissolved in water (50 ml) and this was mixed with dichloroacetic acid (6.19 g, 48 mmol), ferric nitrate nonahydrate (6.46 g, 16 mmol) dissolved in water (30 ml) along with manganese nitrate hexahydrate (2.36 g, 8 mmol) dissolved in water (5 ml). The mixture was stirred for 24 h. The water was removed under reduced pressure and the residue dissolved in a tetrahydrofuran and hexane mixture. The solvent was removed to give an oily residue. This was treated with hexane to remove the oil; the pure compound was obtained by recrystallization from hexane to which several drops of tetrahydrofuran were added. The brown compound melts at 435–436 K.

### Refinement

Carbon-bound H-atoms were placed in calculated positions (C–H = 0.97–0.98 Å) and were included in the refinement in the riding model approximation, with *U_iso_*(H) set to 1.2*U_eq_*(C). The water H-atoms were located in a difference Fourier map, and were refined with distance restraints of O–H = 0.84 (1) Å and H···H 1.37 (1) Å; their U_iso_ parameters were refined.

The manganese(II) atoms are disordered with respect to the iron(III) atoms, with the pair of Mn/Fe atoms occupying the same site. As the occupancy refined to nearly 1:2, the ratio was fixed as exactly 1:2.

For the THF molecule, the O—C distances were restrained to 1.45 (1) Å and the C—C distances to 1.54 (1) Å; the anisotropic displacement parameters of atoms C13, C14, C15 and C16 were restrained to be nearly isotropic.

The final difference map had a peak in the vicinity of O14.

### Crystal data

**Table:**

[Fe_2_Mn(C_2_HCl_2_O_2_)_6_O(C_4_H_8_O)(H_2_O)_2_]	*Z* = 2
*M**_r_* = 1058.34	*F*(000) = 1046
Triclinic, *P*1	*D*_x_ = 1.869 Mg m^−^^3^
Hall symbol: -P 1	Mo *K*α radiation, λ = 0.71073 Å
*a* = 9.380 (1) Å	Cell parameters from 4712 reflections
*b* = 13.316 (1) Å	θ = 2.4–27.4°
*c* = 15.432 (1) Å	µ = 2.01 mm^−^^1^
α = 90.131 (1)°	*T* = 295 K
β = 100.067 (1)°	Block, brown
γ = 97.677 (1)°	0.35 × 0.15 × 0.15 mm
*V* = 1880.1 (2) Å^3^	

### Data collection

**Table:**

Bruker SMART APEX diffractometer	8543 independent reflections
Radiation source: fine-focus sealed tube	5788 reflections with *I* > 2σ(*I*)
graphite	*R*_int_ = 0.025
ω scans	θ_max_ = 27.5°, θ_min_ = 1.3°
Absorption correction: multi-scan (*SADABS*; Sheldrick, 1996)	*h* = −12→12
*T*_min_ = 0.540, *T*_max_ = 0.753	*k* = −17→17
15425 measured reflections	*l* = −20→17

### Refinement

**Table:**

Refinement on *F*^2^	Primary atom site location: structure-invariant direct methods
Least-squares matrix: full	Secondary atom site location: difference Fourier map
*R*[*F*^2^ > 2σ(*F*^2^)] = 0.063	Hydrogen site location: inferred from neighbouring sites
*wR*(*F*^2^) = 0.207	H atoms treated by a mixture of independent and constrained refinement
*S* = 1.02	*w* = 1/[σ^2^(*F*_o_^2^) + (0.1141*P*)^2^ + 3.6071*P*] where *P* = (*F*_o_^2^ + 2*F*_c_^2^)/3
8543 reflections	(Δ/σ)_max_ = 0.001
440 parameters	Δρ_max_ = 1.64 e Å^−^^3^
35 restraints	Δρ_min_ = −0.90 e Å^−^^3^

### Fractional atomic coordinates and isotropic or equivalent isotropic displacement parameters (Å^2^)

**Table:**

	*x*	*y*	*z*	*U*_iso_*/*U*_eq_	Occ. (<1)
Fe1	0.48559 (8)	0.58734 (5)	0.15449 (5)	0.02900 (19)	0.67
Fe2	0.70394 (8)	0.77291 (5)	0.26487 (5)	0.03113 (19)	0.67
Fe3	0.62644 (8)	0.55454 (6)	0.36749 (5)	0.0331 (2)	0.67
Mn1	0.48559 (8)	0.58734 (5)	0.15449 (5)	0.02900 (19)	0.33
Mn2	0.70394 (8)	0.77291 (5)	0.26487 (5)	0.03113 (19)	0.33
Mn3	0.62644 (8)	0.55454 (6)	0.36749 (5)	0.0331 (2)	0.33
Cl1	0.7881 (3)	0.31545 (18)	0.11574 (16)	0.0856 (7)	
Cl2	0.5060 (4)	0.21726 (17)	0.1440 (3)	0.1210 (11)	
Cl3	0.9003 (3)	0.83097 (19)	−0.00937 (16)	0.0953 (8)	
Cl4	0.9893 (3)	0.6422 (2)	0.0556 (2)	0.1111 (10)	
Cl5	0.3394 (5)	0.9362 (2)	0.0396 (2)	0.1385 (14)	
Cl6	0.3033 (4)	0.9569 (2)	0.2189 (3)	0.1291 (12)	
Cl7	0.1673 (3)	0.30107 (17)	0.22744 (18)	0.0944 (8)	
Cl8	0.0953 (3)	0.4052 (2)	0.37391 (18)	0.1134 (11)	
Cl9	0.5161 (3)	0.77820 (18)	0.60071 (13)	0.0888 (7)	
Cl10	0.6888 (3)	0.94612 (19)	0.53350 (18)	0.1011 (9)	
Cl11	1.2099 (2)	0.7122 (2)	0.3483 (2)	0.1044 (9)	
Cl12	1.1217 (3)	0.7937 (2)	0.5016 (2)	0.1191 (11)	
O1	0.5657 (5)	0.4523 (3)	0.1497 (3)	0.0473 (10)	
O2	0.6456 (5)	0.4239 (3)	0.2908 (3)	0.0490 (10)	
O3	0.6341 (4)	0.6367 (3)	0.0718 (3)	0.0449 (9)	
O4	0.7827 (5)	0.7618 (3)	0.1493 (3)	0.0522 (11)	
O5	0.3761 (4)	0.7103 (3)	0.1277 (3)	0.0485 (10)	
O6	0.5320 (4)	0.8361 (3)	0.2003 (3)	0.0532 (11)	
O7	0.3171 (4)	0.5173 (3)	0.2051 (3)	0.0503 (10)	
O8	0.3900 (4)	0.4967 (3)	0.3482 (2)	0.0421 (9)	
O9	0.6494 (6)	0.8139 (3)	0.3790 (3)	0.0556 (11)	
O10	0.5825 (4)	0.6753 (3)	0.4504 (3)	0.0426 (9)	
O11	0.8568 (4)	0.6037 (3)	0.4047 (3)	0.0487 (10)	
O12	0.8968 (4)	0.7428 (3)	0.3279 (3)	0.0523 (11)	
O13	0.6045 (4)	0.6424 (2)	0.2557 (2)	0.0310 (7)	
O14	0.8102 (5)	0.9224 (3)	0.2647 (3)	0.0485 (10)	
O1w	0.3539 (5)	0.5332 (3)	0.0336 (2)	0.0428 (9)	
H11	0.354 (9)	0.4734 (18)	0.015 (3)	0.07 (2)*	
H12	0.336 (7)	0.570 (3)	−0.011 (2)	0.046 (18)*	
O2w	0.6460 (4)	0.4572 (3)	0.4791 (3)	0.0407 (8)	
H21	0.580 (7)	0.408 (4)	0.481 (5)	0.08 (3)*	
H22	0.662 (11)	0.489 (6)	0.528 (3)	0.13 (4)*	
C1	0.6208 (6)	0.4026 (4)	0.2124 (4)	0.0396 (12)	
C2	0.6613 (9)	0.2998 (5)	0.1891 (5)	0.0596 (18)	
H2	0.7080	0.2706	0.2433	0.072*	
C3	0.7428 (6)	0.7024 (4)	0.0862 (3)	0.0364 (11)	
C4	0.8391 (7)	0.7079 (6)	0.0146 (5)	0.0615 (19)	
H4	0.7829	0.6730	−0.0391	0.074*	
C5	0.4141 (6)	0.7987 (4)	0.1553 (4)	0.0425 (13)	
C6	0.3003 (8)	0.8696 (5)	0.1314 (7)	0.079 (3)	
H6	0.2031	0.8299	0.1170	0.095*	
C7	0.3020 (6)	0.4845 (4)	0.2785 (4)	0.0393 (12)	
C8	0.1511 (7)	0.4205 (6)	0.2740 (5)	0.0608 (18)	
H8	0.0783	0.4525	0.2340	0.073*	
C9	0.6017 (6)	0.7685 (4)	0.4403 (4)	0.0374 (11)	
C10	0.5580 (7)	0.8389 (5)	0.5070 (4)	0.0504 (15)	
H10	0.4682	0.8634	0.4777	0.061*	
C11	0.9324 (6)	0.6772 (4)	0.3815 (4)	0.0407 (12)	
C12	1.0941 (7)	0.6917 (6)	0.4260 (5)	0.0584 (17)	
H12a	1.1148	0.6303	0.4580	0.070*	
C13	0.7881 (14)	0.9875 (9)	0.1903 (8)	0.140 (5)	
H13A	0.7012	1.0197	0.1900	0.168*	
H13B	0.7769	0.9488	0.1356	0.168*	
C14	0.9230 (15)	1.0664 (10)	0.2007 (8)	0.143 (5)	
H14A	1.0047	1.0406	0.1816	0.171*	
H14B	0.9039	1.1280	0.1700	0.171*	
C15	0.9457 (18)	1.0811 (8)	0.2983 (8)	0.144 (5)	
H15A	0.8773	1.1229	0.3150	0.173*	
H15B	1.0445	1.1126	0.3213	0.173*	
C16	0.9183 (18)	0.9741 (9)	0.3316 (9)	0.165 (6)	
H16A	1.0069	0.9424	0.3396	0.198*	
H16B	0.8833	0.9744	0.3871	0.198*	

### Atomic displacement parameters (Å^2^)

**Table:**

	*U*^11^	*U*^22^	*U*^33^	*U*^12^	*U*^13^	*U*^23^
Fe1	0.0335 (4)	0.0263 (4)	0.0262 (4)	0.0008 (3)	0.0051 (3)	0.0000 (3)
Fe2	0.0348 (4)	0.0247 (4)	0.0311 (4)	−0.0016 (3)	0.0025 (3)	0.0006 (3)
Fe3	0.0358 (4)	0.0320 (4)	0.0305 (4)	0.0010 (3)	0.0059 (3)	0.0003 (3)
Mn1	0.0335 (4)	0.0263 (4)	0.0262 (4)	0.0008 (3)	0.0051 (3)	0.0000 (3)
Mn2	0.0348 (4)	0.0247 (4)	0.0311 (4)	−0.0016 (3)	0.0025 (3)	0.0006 (3)
Mn3	0.0358 (4)	0.0320 (4)	0.0305 (4)	0.0010 (3)	0.0059 (3)	0.0003 (3)
Cl1	0.0947 (15)	0.0943 (15)	0.0880 (15)	0.0563 (13)	0.0386 (12)	0.0159 (12)
Cl2	0.131 (2)	0.0474 (11)	0.186 (3)	−0.0117 (13)	0.050 (2)	−0.0354 (15)
Cl3	0.1197 (19)	0.0878 (15)	0.0785 (14)	−0.0301 (14)	0.0511 (14)	0.0132 (12)
Cl4	0.0769 (14)	0.120 (2)	0.155 (3)	0.0284 (14)	0.0592 (17)	−0.0058 (19)
Cl5	0.214 (4)	0.104 (2)	0.098 (2)	0.074 (2)	−0.009 (2)	0.0416 (17)
Cl6	0.134 (2)	0.0947 (19)	0.185 (3)	0.0527 (18)	0.072 (2)	−0.010 (2)
Cl7	0.1038 (17)	0.0655 (13)	0.1057 (18)	−0.0294 (12)	0.0273 (14)	−0.0193 (12)
Cl8	0.0954 (17)	0.150 (3)	0.0903 (17)	−0.0524 (17)	0.0554 (14)	−0.0208 (16)
Cl9	0.145 (2)	0.0797 (14)	0.0520 (11)	0.0171 (14)	0.0442 (12)	−0.0051 (10)
Cl10	0.1105 (18)	0.0784 (14)	0.1083 (18)	−0.0314 (13)	0.0369 (15)	−0.0541 (14)
Cl11	0.0527 (11)	0.142 (2)	0.132 (2)	0.0308 (13)	0.0393 (13)	0.0530 (18)
Cl12	0.0953 (18)	0.121 (2)	0.116 (2)	−0.0117 (16)	−0.0304 (16)	−0.0442 (18)
O1	0.072 (3)	0.033 (2)	0.037 (2)	0.0182 (19)	0.0018 (19)	−0.0010 (16)
O2	0.074 (3)	0.042 (2)	0.034 (2)	0.018 (2)	0.0087 (19)	−0.0006 (17)
O3	0.044 (2)	0.053 (2)	0.035 (2)	−0.0111 (18)	0.0133 (16)	−0.0022 (17)
O4	0.063 (3)	0.046 (2)	0.045 (2)	−0.013 (2)	0.020 (2)	−0.0057 (19)
O5	0.048 (2)	0.038 (2)	0.055 (3)	0.0091 (18)	−0.0061 (19)	0.0007 (18)
O6	0.043 (2)	0.033 (2)	0.075 (3)	0.0012 (17)	−0.010 (2)	0.004 (2)
O7	0.043 (2)	0.065 (3)	0.035 (2)	−0.0156 (19)	0.0043 (17)	0.0022 (19)
O8	0.039 (2)	0.052 (2)	0.033 (2)	−0.0039 (17)	0.0066 (16)	0.0045 (17)
O9	0.088 (3)	0.033 (2)	0.048 (2)	−0.005 (2)	0.028 (2)	−0.0066 (18)
O10	0.055 (2)	0.036 (2)	0.039 (2)	0.0071 (17)	0.0136 (18)	−0.0020 (16)
O11	0.036 (2)	0.049 (2)	0.059 (3)	0.0001 (18)	0.0065 (18)	0.012 (2)
O12	0.039 (2)	0.046 (2)	0.063 (3)	−0.0021 (18)	−0.0071 (19)	0.013 (2)
O13	0.0362 (18)	0.0248 (16)	0.0294 (17)	−0.0007 (13)	0.0021 (14)	0.0022 (13)
O14	0.055 (2)	0.031 (2)	0.054 (3)	−0.0073 (17)	0.003 (2)	0.0038 (17)
O1w	0.051 (2)	0.045 (2)	0.0296 (19)	0.0013 (19)	0.0038 (17)	−0.0049 (17)
O2w	0.046 (2)	0.043 (2)	0.032 (2)	0.0025 (18)	0.0067 (16)	0.0062 (17)
C1	0.049 (3)	0.035 (3)	0.037 (3)	0.007 (2)	0.013 (2)	0.000 (2)
C2	0.101 (5)	0.039 (3)	0.048 (4)	0.032 (3)	0.020 (4)	0.004 (3)
C3	0.040 (3)	0.038 (3)	0.032 (3)	0.001 (2)	0.011 (2)	0.002 (2)
C4	0.052 (4)	0.080 (5)	0.052 (4)	−0.019 (3)	0.027 (3)	−0.009 (3)
C5	0.036 (3)	0.039 (3)	0.051 (3)	0.004 (2)	0.005 (2)	0.009 (3)
C6	0.046 (4)	0.038 (3)	0.145 (8)	0.011 (3)	−0.010 (4)	0.016 (4)
C7	0.030 (2)	0.044 (3)	0.042 (3)	−0.005 (2)	0.008 (2)	−0.005 (2)
C8	0.047 (3)	0.067 (4)	0.065 (4)	−0.015 (3)	0.016 (3)	0.000 (3)
C9	0.040 (3)	0.034 (3)	0.036 (3)	0.002 (2)	0.001 (2)	−0.005 (2)
C10	0.060 (4)	0.045 (3)	0.047 (3)	0.006 (3)	0.013 (3)	−0.012 (3)
C11	0.033 (3)	0.043 (3)	0.045 (3)	0.006 (2)	0.004 (2)	−0.004 (2)
C12	0.038 (3)	0.063 (4)	0.069 (4)	0.002 (3)	−0.002 (3)	0.012 (3)
C13	0.154 (9)	0.108 (7)	0.138 (8)	−0.014 (7)	−0.003 (7)	0.030 (7)
C14	0.152 (8)	0.112 (7)	0.154 (9)	−0.030 (6)	0.039 (7)	0.032 (7)
C15	0.188 (9)	0.082 (6)	0.136 (8)	−0.059 (6)	0.013 (7)	0.009 (6)
C16	0.179 (9)	0.122 (8)	0.167 (9)	−0.020 (7)	−0.010 (8)	0.010 (7)

### Geometric parameters (Å, °)

**Table:**

Fe1—O13	1.841 (3)	O7—C7	1.239 (7)
Fe1—O7	2.004 (4)	O8—C7	1.232 (7)
Fe1—O1	2.045 (4)	O9—C9	1.241 (7)
Fe1—O5	2.052 (4)	O10—C9	1.244 (6)
Fe1—O3	2.092 (4)	O11—C11	1.222 (7)
Fe1—O1w	2.116 (4)	O12—C11	1.241 (7)
Fe2—O13	1.852 (3)	O14—C16	1.419 (9)
Fe2—O12	1.991 (4)	O14—C13	1.442 (8)
Fe2—O9	2.012 (4)	O1w—H11	0.85 (1)
Fe2—O6	2.026 (4)	O1w—H12	0.85 (1)
Fe2—O4	2.058 (4)	O2w—H21	0.85 (1)
Fe2—O14	2.104 (4)	O2w—H22	0.85 (1)
Fe3—O13	2.082 (3)	C1—C2	1.528 (8)
Fe3—O2	2.146 (4)	C2—H2	0.98
Fe3—O11	2.148 (4)	C3—C4	1.541 (8)
Fe3—O2w	2.155 (4)	C4—H4	0.98
Fe3—O10	2.178 (4)	C5—C6	1.516 (8)
Fe3—O8	2.216 (4)	C6—H6	0.98
Cl1—C2	1.773 (8)	C7—C8	1.542 (8)
Cl2—C2	1.740 (9)	C8—H8	0.98
Cl3—C4	1.725 (8)	C9—C10	1.534 (8)
Cl4—C4	1.781 (9)	C10—H10	0.98
Cl5—C6	1.740 (10)	C11—C12	1.538 (8)
Cl6—C6	1.774 (10)	C12—H12a	0.98
Cl7—C8	1.779 (8)	C13—C14	1.519 (9)
Cl8—C8	1.717 (7)	C13—H13A	0.97
Cl9—C10	1.736 (7)	C13—H13B	0.97
Cl10—C10	1.751 (7)	C14—C15	1.493 (9)
Cl11—C12	1.751 (8)	C14—H14A	0.97
Cl12—C12	1.752 (8)	C14—H14B	0.97
O1—C1	1.250 (7)	C15—C16	1.521 (9)
O2—C1	1.217 (7)	C15—H15A	0.97
O3—C3	1.240 (6)	C15—H15B	0.97
O4—C3	1.226 (7)	C16—H16A	0.97
O5—C5	1.236 (7)	C16—H16B	0.97
O6—C5	1.240 (7)		
			
O13—Fe1—O7	100.17 (16)	Cl2—C2—Cl1	111.0 (4)
O13—Fe1—O1	98.93 (16)	C1—C2—H2	108.3
O7—Fe1—O1	89.64 (19)	Cl2—C2—H2	108.3
O13—Fe1—O5	95.75 (16)	Cl1—C2—H2	108.3
O7—Fe1—O5	89.80 (19)	O4—C3—O3	128.6 (5)
O1—Fe1—O5	165.17 (16)	O4—C3—C4	117.0 (5)
O13—Fe1—O3	94.81 (15)	O3—C3—C4	114.4 (5)
O7—Fe1—O3	164.61 (16)	C3—C4—Cl3	112.5 (5)
O1—Fe1—O3	84.53 (18)	C3—C4—Cl4	106.3 (5)
O5—Fe1—O3	92.22 (18)	Cl3—C4—Cl4	110.5 (4)
O13—Fe1—O1w	176.02 (16)	C3—C4—H4	109.1
O7—Fe1—O1w	83.07 (16)	Cl3—C4—H4	109.1
O1—Fe1—O1w	83.33 (17)	Cl4—C4—H4	109.1
O5—Fe1—O1w	81.89 (17)	O5—C5—O6	128.3 (5)
O3—Fe1—O1w	82.11 (16)	O5—C5—C6	115.3 (5)
O13—Fe2—O12	98.58 (16)	O6—C5—C6	116.4 (6)
O13—Fe2—O9	97.57 (16)	C5—C6—Cl5	107.9 (6)
O12—Fe2—O9	90.9 (2)	C5—C6—Cl6	111.4 (6)
O13—Fe2—O6	94.49 (16)	Cl5—C6—Cl6	109.1 (4)
O12—Fe2—O6	166.89 (17)	C5—C6—H6	109.5
O9—Fe2—O6	88.4 (2)	Cl5—C6—H6	109.5
O13—Fe2—O4	94.61 (16)	Cl6—C6—H6	109.5
O12—Fe2—O4	87.6 (2)	O8—C7—O7	128.3 (5)
O9—Fe2—O4	167.81 (17)	O8—C7—C8	121.0 (5)
O6—Fe2—O4	90.4 (2)	O7—C7—C8	110.7 (5)
O13—Fe2—O14	175.47 (16)	C7—C8—Cl8	114.3 (5)
O12—Fe2—O14	84.51 (17)	C7—C8—Cl7	105.4 (5)
O9—Fe2—O14	85.66 (17)	Cl8—C8—Cl7	110.5 (4)
O6—Fe2—O14	82.38 (16)	C7—C8—H8	108.8
O4—Fe2—O14	82.17 (17)	Cl8—C8—H8	108.8
O13—Fe3—O2	91.27 (14)	Cl7—C8—H8	108.8
O13—Fe3—O11	94.02 (15)	O9—C9—O10	127.3 (5)
O2—Fe3—O11	96.35 (18)	O9—C9—C10	113.6 (5)
O13—Fe3—O2w	177.25 (15)	O10—C9—C10	119.1 (5)
O2—Fe3—O2w	86.15 (16)	C9—C10—Cl9	113.7 (4)
O11—Fe3—O2w	87.23 (16)	C9—C10—Cl10	111.5 (4)
O13—Fe3—O10	92.63 (14)	Cl9—C10—Cl10	111.7 (4)
O2—Fe3—O10	172.53 (16)	C9—C10—H10	106.5
O11—Fe3—O10	89.72 (17)	Cl9—C10—H10	106.5
O2w—Fe3—O10	89.82 (15)	Cl10—C10—H10	106.5
O13—Fe3—O8	93.57 (14)	O11—C11—O12	129.2 (5)
O2—Fe3—O8	85.85 (17)	O11—C11—C12	115.5 (5)
O11—Fe3—O8	172.04 (15)	O12—C11—C12	115.3 (5)
O2w—Fe3—O8	85.28 (15)	C11—C12—Cl11	111.3 (5)
O10—Fe3—O8	87.56 (15)	C11—C12—Cl12	107.9 (5)
C1—O1—Fe1	128.2 (4)	Cl11—C12—Cl12	111.4 (4)
C1—O2—Fe3	134.2 (4)	C11—C12—H12a	108.7
C3—O3—Fe1	129.1 (3)	Cl11—C12—H12a	108.7
C3—O4—Fe2	130.9 (4)	Cl12—C12—H12a	108.7
C5—O5—Fe1	128.3 (4)	O14—C13—C14	106.2 (8)
C5—O6—Fe2	132.2 (4)	O14—C13—H13A	110.5
C7—O7—Fe1	134.5 (4)	C14—C13—H13A	110.5
C7—O8—Fe3	128.0 (3)	O14—C13—H13B	110.5
C9—O9—Fe2	135.5 (4)	C14—C13—H13B	110.5
C9—O10—Fe3	128.8 (4)	H13A—C13—H13B	108.7
C11—O11—Fe3	130.0 (4)	C15—C14—C13	97.9 (9)
C11—O12—Fe2	132.6 (4)	C15—C14—H14A	112.2
Fe1—O13—Fe2	123.79 (18)	C13—C14—H14A	112.2
Fe1—O13—Fe3	118.50 (16)	C15—C14—H14B	112.2
Fe2—O13—Fe3	117.70 (17)	C13—C14—H14B	112.2
C16—O14—C13	108.7 (7)	H14A—C14—H14B	109.8
C16—O14—Fe2	127.9 (5)	C14—C15—C16	103.8 (10)
C13—O14—Fe2	123.3 (5)	C14—C15—H15A	111.0
Fe1—O1w—H11	121 (4)	C16—C15—H15A	111.0
Fe1—O1w—H12	124 (4)	C14—C15—H15B	111.0
H11—O1w—H12	107.6 (17)	C16—C15—H15B	111.0
Fe3—O2w—H21	119 (6)	H15A—C15—H15B	109.0
Fe3—O2w—H22	114 (7)	O14—C16—C15	104.4 (8)
H21—O2w—H22	107.5 (18)	O14—C16—H16A	110.9
O2—C1—O1	129.0 (5)	C15—C16—H16A	110.9
O2—C1—C2	114.4 (5)	O14—C16—H16B	110.9
O1—C1—C2	116.5 (5)	C15—C16—H16B	110.9
C1—C2—Cl2	110.8 (5)	H16A—C16—H16B	108.9
C1—C2—Cl1	110.0 (5)		
			
O13—Fe1—O1—C1	30.2 (5)	O3—Fe1—O13—Fe3	−134.8 (2)
O7—Fe1—O1—C1	−70.1 (5)	O12—Fe2—O13—Fe1	−135.3 (2)
O5—Fe1—O1—C1	−157.9 (6)	O9—Fe2—O13—Fe1	132.7 (2)
O3—Fe1—O1—C1	124.2 (5)	O6—Fe2—O13—Fe1	43.7 (3)
O1w—Fe1—O1—C1	−153.2 (5)	O4—Fe2—O13—Fe1	−47.0 (3)
O13—Fe3—O2—C1	−14.5 (6)	O12—Fe2—O13—Fe3	45.9 (2)
O11—Fe3—O2—C1	−108.7 (6)	O9—Fe2—O13—Fe3	−46.1 (2)
O2w—Fe3—O2—C1	164.6 (6)	O6—Fe2—O13—Fe3	−135.1 (2)
O8—Fe3—O2—C1	79.0 (6)	O4—Fe2—O13—Fe3	134.2 (2)
O13—Fe1—O3—C3	−16.7 (5)	O2—Fe3—O13—Fe1	43.2 (2)
O7—Fe1—O3—C3	176.6 (6)	O11—Fe3—O13—Fe1	139.6 (2)
O1—Fe1—O3—C3	−115.2 (5)	O10—Fe3—O13—Fe1	−130.5 (2)
O5—Fe1—O3—C3	79.3 (5)	O8—Fe3—O13—Fe1	−42.8 (2)
O1w—Fe1—O3—C3	160.8 (5)	O2—Fe3—O13—Fe2	−138.0 (2)
O13—Fe2—O4—C3	18.4 (6)	O11—Fe3—O13—Fe2	−41.5 (2)
O12—Fe2—O4—C3	116.8 (6)	O10—Fe3—O13—Fe2	48.4 (2)
O9—Fe2—O4—C3	−160.3 (8)	O8—Fe3—O13—Fe2	136.1 (2)
O6—Fe2—O4—C3	−76.2 (6)	O12—Fe2—O14—C13	139.5 (9)
O14—Fe2—O4—C3	−158.4 (6)	O9—Fe2—O14—C13	−129.2 (9)
O13—Fe1—O5—C5	19.9 (5)	O6—Fe2—O14—C13	−40.2 (9)
O7—Fe1—O5—C5	120.1 (5)	O4—Fe2—O14—C13	51.2 (9)
O1—Fe1—O5—C5	−152.1 (7)	Fe3—O2—C1—O1	−4.6 (10)
O3—Fe1—O5—C5	−75.2 (5)	Fe3—O2—C1—C2	176.5 (5)
O1w—Fe1—O5—C5	−156.9 (5)	Fe1—O1—C1—C2	175.7 (4)
O13—Fe2—O6—C5	−13.1 (6)	O2—C1—C2—Cl2	114.7 (6)
O12—Fe2—O6—C5	162.4 (8)	O1—C1—C2—Cl2	−64.4 (7)
O9—Fe2—O6—C5	−110.6 (6)	O2—C1—C2—Cl1	−122.3 (5)
O4—Fe2—O6—C5	81.6 (6)	O1—C1—C2—Cl1	58.7 (7)
O14—Fe2—O6—C5	163.6 (6)	Fe2—O4—C3—C4	−172.8 (5)
O13—Fe1—O7—C7	−23.4 (6)	Fe1—O3—C3—O4	−7.7 (9)
O1—Fe1—O7—C7	75.7 (6)	Fe1—O3—C3—C4	171.8 (4)
O5—Fe1—O7—C7	−119.2 (6)	O4—C3—C4—Cl3	−41.7 (8)
O3—Fe1—O7—C7	143.2 (6)	O3—C3—C4—Cl3	138.7 (5)
O1w—Fe1—O7—C7	159.0 (6)	O4—C3—C4—Cl4	79.4 (6)
O13—Fe3—O8—C7	28.5 (5)	O3—C3—C4—Cl4	−100.2 (5)
O2—Fe3—O8—C7	−62.6 (5)	Fe1—O5—C5—C6	−173.3 (5)
O2w—Fe3—O8—C7	−149.0 (5)	Fe2—O6—C5—C6	169.0 (5)
O10—Fe3—O8—C7	120.9 (5)	O5—C5—C6—Cl5	−97.9 (6)
O13—Fe2—O9—C9	18.2 (6)	O6—C5—C6—Cl5	82.8 (7)
O12—Fe2—O9—C9	−80.6 (6)	O5—C5—C6—Cl6	142.3 (5)
O6—Fe2—O9—C9	112.5 (6)	O6—C5—C6—Cl6	−37.0 (8)
O4—Fe2—O9—C9	−163.2 (8)	Fe3—O8—C7—O7	−13.7 (9)
O14—Fe2—O9—C9	−165.0 (6)	Fe3—O8—C7—C8	165.3 (4)
O13—Fe3—O10—C9	−25.7 (5)	Fe1—O7—C7—C8	−169.9 (5)
O11—Fe3—O10—C9	68.3 (5)	O8—C7—C8—Cl8	20.3 (8)
O2w—Fe3—O10—C9	155.5 (5)	O7—C7—C8—Cl8	−160.5 (5)
O8—Fe3—O10—C9	−119.2 (5)	O8—C7—C8—Cl7	−101.2 (6)
O13—Fe3—O11—C11	17.7 (5)	O7—C7—C8—Cl7	78.0 (6)
O2—Fe3—O11—C11	109.4 (5)	Fe2—O9—C9—C10	−171.7 (5)
O2w—Fe3—O11—C11	−164.8 (5)	Fe3—O10—C9—O9	1.0 (9)
O10—Fe3—O11—C11	−74.9 (5)	Fe3—O10—C9—C10	178.3 (4)
O13—Fe2—O12—C11	−30.9 (6)	O9—C9—C10—Cl9	−171.3 (5)
O9—Fe2—O12—C11	66.9 (6)	O10—C9—C10—Cl9	11.0 (7)
O6—Fe2—O12—C11	153.6 (8)	O9—C9—C10—Cl10	−44.0 (7)
O4—Fe2—O12—C11	−125.2 (6)	O10—C9—C10—Cl10	138.4 (5)
O14—Fe2—O12—C11	152.4 (6)	Fe3—O11—C11—C12	176.9 (4)
O7—Fe1—O13—Fe2	−137.2 (2)	Fe2—O12—C11—C12	−170.4 (4)
O1—Fe1—O13—Fe2	131.6 (2)	O11—C11—C12—Cl11	131.1 (5)
O5—Fe1—O13—Fe2	−46.3 (2)	O12—C11—C12—Cl11	−49.9 (7)
O3—Fe1—O13—Fe2	46.4 (2)	O11—C11—C12—Cl12	−106.4 (6)
O7—Fe1—O13—Fe3	41.6 (2)	O12—C11—C12—Cl12	72.7 (6)
O1—Fe1—O13—Fe3	−49.6 (2)	Fe2—O14—C13—C14	−157.0 (8)
O5—Fe1—O13—Fe3	132.5 (2)	Fe2—O14—C16—C15	−175.9 (8)

### Hydrogen-bond geometry (Å, °)

**Table:**

*D*—H···*A*	*D*—H	H···*A*	*D*···*A*	*D*—H···*A*
O1w—H11···O3^i^	0.85 (1)	2.01 (3)	2.809 (6)	158 (5)
O2w—H22···O8^ii^	0.85 (1)	2.06 (4)	2.821 (5)	149 (7)
O2W—H21···O10^ii^	0.84 (6)	2.19 (7)	2.950 (6)	150 (6)
O1W—H12···Cl1^i^	0.85 (3)	2.47 (4)	3.288 (4)	160 (6)

Symmetry codes: (i) −*x*+1, −*y*+1, −*z*; (ii) −*x*+1, −*y*+1, −*z*+1.
